# Molecular phylogeny and taxonomy of the remarkable genus *Leptoporus* (Polyporales, Basidiomycota) with description of a new species from Southwest China

**DOI:** 10.3389/fcimb.2022.1116035

**Published:** 2023-01-23

**Authors:** Shun Liu, Yi-Fei Sun, Xing Ji, Chang-Ge Song, Tai-Min Xu, Bao-Kai Cui

**Affiliations:** Institute of Microbiology, School of Ecology and Nature Conservation, Beijing Forestry University, Beijing, China

**Keywords:** brown-rot fungi, Irpicaceae, macro-fungi, multi-gene phylogeny, taxonomy

## Abstract

*Leptoporus* is a rare and remarkable genus, mainly occurring in coniferous forests in the Northern Hemisphere. Recent phylogenetic studies showed that *Leptoporus* belongs to Irpicaceae in the phlebioid clade. It is worth noting that most species in the phlebioid clade can cause white-rot decay, except for the *Leptoporus* species, which can cause a brown-rot decay. In this study, we performed phylogenetic and taxonomic studies of *Leptoporus* and related genera. Molecular phylogenetic analyses were conducted based on sequences from multiple loci including the internal transcribed spacer (ITS) regions, the large subunit of nuclear ribosomal RNA gene (nLSU), the largest subunit of RNA polymerase II gene (*RPB1*), the second largest subunit of RNA polymerase II gene (*RPB2*), and the translation elongation factor 1-α gene (*TEF1*). Combined with morphological characteristics, a new species, *Leptoporus submollis* sp. nov., is discovered and illustrated from Southwest China.

## Introduction

Irpicaceae Spirin & Zmitr. was proposed by Spirin (2003) with *Irpex* Fr. as type genus. The great majority of the species in Irpicaceae, even in the phlebioid clade, can cause a white rot, except for *Leptoporus mollis* (Pers.) Quél., which causes a brown rot (Gilbertson and Ryvarden, 1986; Chen et al., 2021). This makes *Leptoporus* a remarkable genus, which has attracted many mycologists’ attention.

*Leptoporus* Quél. was established by Quél (1886), with *L. mollis* as type species, which was described as causing a brown rot on dead conifers and mainly distributed in the Northern Hemisphere (North America, Europe, and Asia) (Gilbertson and Ryvarden, 1986; Ryvarden and Gilbertson, 1993; Núñez and Ryvarden, 2001; Yu et al., 2004; Volobuev, 2019). In North America, *L. mollis* has been reported in boreal coniferous forests (Gilbertson and Ryvarden, 1986). In Europe, this species was considered as a rare species and needs to be protected (Ryvarden and Gilbertson, 1993; Volobuev, 2019). In Asia, this species has been reported from China and Japan and was also considered as a rare species (Núñez and Ryvarden, 2001; Yu et al., 2004). Previously, *Leptoporus* was placed in Polyporaceae Fr. ex Corda (Yu et al., 2004; Kirk et al., 2008). Subsequently, some phylogenetic studies showed that *Leptoporus* was embedded in the phlebioid clade (Binder et al., 2005; Lindner and Banik, 2008; Binder et al., 2013). In recent years, *Leptoporus* has been proven to belong to Irpicaceae and was closely related to *Ceriporia* Donk (Justo et al., 2017; Chen et al., 2021). Currently, although the databases Index Fungorum (http://www.indexfungorum.org/) and MycoBank (https://www.mycobank.org/) still record some *Leptoporus* species, only one species, *L. mollis*, is accepted in recent studies (Lindner and Banik, 2008; He et al., 2019; Chen et al., 2020; Chen et al., 2021).

During investigations on the diversity of polypores in the Hengduan Mountains of Southwest China, one undescribed species of *Leptoporus* was discovered. To confirm the affinity of the undescribed species corresponding to *Leptoporus*, phylogenetic analyses of Irpicaceae were carried out based on the combined sequences datasets of ITS+nLSU and ITS+nLSU+*RPB1*+*RPB2*+*TEF1*.

## Materials and methods

### Morphological studies

The examined specimens were mostly deposited at the herbarium of the Institute of Microbiology, Beijing Forestry University, China (BJFC), and some specimens were deposited at the Institute of Applied Ecology, Chinese Academy of Sciences, China (IFP). Macromorphological descriptions were based on the field notes and measurements of herbarium specimens. Special color terms followed Petersen (1996). Micromorphological data were obtained from the dried specimens and observed under a light microscope following Ji et al. (2022) and Sun et al. (2022). Sections were studied at a magnification up to ×1,000 using a Nikon Eclipse 80i microscope and phase-contrast illumination (Nikon, Tokyo, Japan). Drawings were made with the aid of a drawing tube. Microscopic features, measurements, and drawings were made from slide preparations stained with Cotton Blue and Melzer’s reagent. Spores were measured from sections cut from the tubes. To present variations in the size of basidiospores, 5% of measurements were excluded from each end of the range and extreme values are given in parentheses.

In the text, the following abbreviations were used: IKI, Melzer’s reagent; IKI–, neither amyloid nor dextrinoid; KOH, 5% potassium hydroxide; CB, Cotton Blue; CB–, acyanophilous; L, mean spore length (arithmetic average of all spores); W, mean spore width (arithmetic average of all spores); Q, variation in the L/W ratios between the specimens studied; n (a/b), number of spores (a) measured from given number (b) of specimens.

### Molecular studies and phylogenetic analysis

A cetyl trimethylammonium bromide (CTAB) rapid plant genome extraction kit-DN14 (Aidlab Biotechnologies Co., Ltd., Beijing, China) was used to extract total genomic DNA from dried specimens, and the polymerase chain reaction (PCR) was performed according to the manufacturer’s instructions with some modifications as described by Cui et al. (2019) and Shen et al. (2019). The internal transcribed spacer (ITS) regions were amplified with primer pairs ITS5 and ITS4 (White et al., 1990). The large subunit of nuclear ribosomal RNA gene (nLSU) regions were amplified with primer pairs LR0R and LR7 (http://www.biology.duke.edu/fungi/mycolab/primers.htm). *RPB1* was amplified with primer pairs RPB1-Af and RPB1-Cr (Matheny et al., 2002). *RPB2* was amplified with primer pairs fRPB2-f5F and bRPB2-7.1R (Matheny, 2005). Part of *TEF1* was amplified with primer pairs EF1-983F and EF1-1567R (Rehner, 2001).

The PCR cycling schedule for ITS and *TEF1* included an initial denaturation at 95°C for 3 min, followed by 35 cycles at 94°C for 40 s, 54°C for ITS, 55°C for *TEF1* for 45 s, 72°C for 1 min, and a final extension at 72°C for 10 min. The PCR cycling schedule for nLSU included an initial denaturation at 94°C for 1 min, followed by 35 cycles at 94°C for 30 s, 51°C for 1 min, 72°C for 1.5 min, and a final extension at 72°C for 10 min. The PCR cycling schedule for *RPB1* and *RPB2* included an initial denaturation at 94°C for 2 min, followed by 10 cycles at 94°C for 40 s, 60°C for 40 s, and 72°C for 2 min, then followed by 37 cycles at 94°C for 45 s, 55°C–57°C for 1.5 min, 72°C for 2 min, and a final extension of 72°C for 10 min. The PCR products were purified and sequenced at Beijing Genomics Institute (BGI), China, with the same primers. All newly generated sequences were deposited at GenBank (**Table 1**).

**Table 1:** A list of species, specimens, and GenBank accession number of sequences used for phylogenetic analyses in this study.

Species	Sample no.	Locality	GenBank accessions					References
			ITS	nLSU	*RPB1*	*RPB2*	*TEF1*	
Byssomerulius corium	FCUG 2701	Russia	MZ636931	GQ470630	MZ748415	OK136068	MZ913668	Wu et al., 2010; Chen et al., 2021
*Byssomerulius corium*	Wu 1207-55	China	MZ636932	MZ637096	—	—	—	Chen et al., 2021
*Byssomerulius corium*	FP-102382	USA	KP135007	KP135230	KP134802	KP134921		Floudas and Hibbett, 2015
*Ceriporia bubalinomarginata*	Dai 11327	China	JX623953	JX644045	—	—	—	Jia et al., 2014
*Ceriporia bubalinomarginata*	Dai 12499	China	JX623954	JX644044	—	—	—	Jia et al., 2014
*Ceriporia viridans*	Spirin 5909	Finland	KX236481	KX236481	—	—	—	Spirin et al., 2016
*Ceriporia viridans*	Miettinen 1170	Netherlands	KX752600	KX752600	—	—	—	Miettinen et al., 2016
*Crystallicutis* cf. *serpens*	Wu 1608-130	China	MZ636946	MZ637108	—	—	—	Chen et al., 2021
*Crystallicutis* cf. *serpens*	Wu 1608-81	China	MZ636947	MZ637109	MZ748435	OK136094	MZ913699	Chen et al., 2021
*Crystallicutis serpens*	HHB-15692	USA	KP135031	KP135200	KP134785	KP134914	—	Floudas and Hibbett, 2015
*Cytidiella albida*	GB-1833	Spain	KY948748	KY948889	KY948960	OK136069	MZ913675	Justo et al., 2017; Chen et al., 2021
*Cytidiella albomarginata*	Wei 18-474	China	MZ636948	MZ637110	MZ748429	OK136070	MZ913678	Chen et al., 2021
*Cytidiella albomarginata*	Wu 0108-86	China	MZ636949	MZ637111	MZ748430	OK136071	MZ913677	Chen et al., 2021
*Cytidiella albomellea*	FP-102339	USA	MZ636950	MZ637112	MZ748431	—	—	Chen et al., 2021
*Cytidiella nitidula*	T-407	USA	KY948747	MZ637113	KY948961	OK136072	MZ913676	Justo et al., 2017; Chen et al., 2021
*Efibula gracilis*	FD-455	USA	KP135027	MZ637116	KP134804	OK136077	MZ913679	Floudas and Hibbett, 2015; Chen et al., 2021
*Efibula intertexta*	Wu 1707-93	China	MZ636953	MZ637117	MZ748416	OK136085	—	Chen et al., 2021
*Efibula intertexta*	Wu 1707-96	China	MZ636954	MZ637118	MZ748417	OK136086	—	Chen et al., 2021
*Efibula matsuensis*	Wu 1011-18	China	MZ636956	MZ637119	MZ748418	OK136078	MZ913680	Chen et al., 2021
*Efibula tropica*	Wei 18-149	China	MZ636967	MZ637129	MZ748419	OK136079	MZ913681	Chen et al., 2021
*Efibula tropica*	Chen 3596	China (Taiwan)	MZ636966	MZ637128	—	—	—	Chen et al., 2021
*Efibula yunnanensis*	Wu 880515-1	China	MZ636977	GQ470672	MZ748420	OK136080	MZ913682	Wu et al., 2010; Chen et al., 2021
*Gloeoporus orientalis*	Wei 16-485	China	MZ636980	MZ637141	MZ748443	OK136095	MZ913709	Chen et al., 2021
*Gloeoporus pannocinctus*	L-15726	USA	KP135060	KP135214	KP134867	KP134973	—	Floudas and Hibbett, 2015
*Irpex flavus*	Wu 0705-1	China	MZ636988	MZ637149	MZ748432	OK136087	MZ913683	Chen et al., 2021
*Irpex flavus*	Wu 0705-2	China	MZ636989	MZ637150	—	—	—	Chen et al., 2021
*Irpex hydnoides*	KUC 20121109-01	South Korea	KJ668510	KJ668362	—	—	—	Jang et al., 2016
*Irpex laceratus*	WHC 1372	China	MZ636990	MZ637151	—	—	—	Chen et al., 2021
*Irpex lacteus*	DO 421	Sweden	JX109852	JX109852	—	JX109882		Binder et al., 2013
*Irpex lacteus*	FD-9	USA	KP135026	KP135224	KP134806	—	—	Floudas and Hibbett, 2015
*,Irpex latemarginatus*	FP-55521-T	USA	KP135024	KP135202	KP134805	KP134915	—	Floudas and Hibbett, 2015
*Irpex latemarginatus*	Dai 7165	China	KY131834	KY131893	—	—	—	Wu et al., 2017
*Irpex lenis*	Wu 1608-14	China	MZ636991	MZ637152	MZ748434	—	MZ913685	Chen et al., 2021
*Irpex rosettiformis*	Meijer 3729	Brazil	JN649346	JN649346	—	JX109875	JX109904	Sjökvist et al., 2012; Binder et al., 2013
*Irpex* sp.	Wu 910807-35	China	MZ636994	GQ470627	MZ748433	OK136088	MZ913684	Wu et al., 2010; Chen et al., 2021
*Leptoporus mollis*	LE BIN 3849	Russia	MG735341		—	—	—	Psurtseva, 2010
*Leptoporus mollis*	Dai 21062	Belarus	**MW377302**	**MW377381**	—	**MW337062**	**MW337129**	Present study
*Leptoporus mollis*	JV 12117	USA	**MW377303**		—	—	—	Present study
*Leptoporus mollis*	RLG-7163	USA	KY948794	MZ637155	KY948956	OK136101	MZ913693	Justo et al., 2017; Chen et al., 2021
*Leptoporus submollis*	Cui 17584	China	**MW377305**	**MW377383**	**MW337195**	**MW337064**	**MW337131**	Present study
*Leptoporus submollis*	Cui 17514	China	**MW377304**	**MW377382**	**MW337194**	**MW337063**	**MW337130**	Present study
*Leptoporus submollis*	Cui 18379	China	**ON468433**	**ON468245**	**ON468447**	**ON468449**	**ON468451**	Present study
*Leptoporus submollis*	Dai 20182	China	**ON468434**	**ON468246**	**ON468448**	**ON468450**	**ON468452**	Present study
*Meruliopsis albostramineus*	HHB 10729	USA	KP135051	KP135229	KP134787	—	—	Floudas and Hibbett, 2015
*Meruliopsis crassitunicata*	CHWC 1506-46	China	LC427010	LC427034	—	—	—	Chen et al., 2020
*Meruliopsis leptocystidiata*	Wu 1708-43	China	LC427013	LC427033	LC427070	—	—	Chen et al., 2020
*Meruliopsis parvispora*	Wu 1209-58	China	LC427017	LC427039	LC427065	—	—	Chen et al., 2020
*Meruliopsis taxicola*	GC 1704-60	China	LC427028	LC427050	LC427063	—	—	Chen et al., 2020
*Phanerochaete albida*	GC 1407-14	China	MZ422788	MZ637179	MZ748384	OK136013	MZ913704	Chen et al., 2021
*Phanerochaete alnea*	FP-151125	USA	KP135177	MZ637181	MZ748385	OK136014	MZ913641	Floudas and Hibbett, 2015; Chen et al., 2021
*Phanerochaetella angustocystidiata*	Wu 9606-39	China	MZ637020	GQ470638	MZ748422	OK136082	MZ913687	Wu et al., 2010; Chen et al., 2021
*Phanerochaetella angustocystidiata*	Wu 1109-56	China	MZ637019	MZ637227	MZ748421	OK136081	MZ913686	Chen et al., 2021
*Phanerochaetella exilis*	HHB-6988	USA	KP135001	KP135236	KP134799	KP134918	—	Floudas and Hibbett, 2015
*Phanerochaetella formosana*	Chen 479	China	MZ637023	GQ470650	MZ748424	OK136084	MZ913718	Wu et al., 2010; Chen et al., 2021
*Phanerochaetella leptoderma*	Chen 1362	China	MZ637025	GQ470646	MZ748423	OK136083	MZ913689	Wu et al., 2010; Chen et al., 2021
*Phanerochaetella* sp.	HHB-11463	USA	KP134994	KP135235	KP134797	KP134892	—	Floudas and Hibbett, 2015
*Phanerochaetella* sp.	HHB-18104	New Zealand	KP135003	KP135254	KP134798	KP134917	—	Floudas and Hibbett, 2015
*Phanerochaetella xerophila*	HHB-8509	USA	KP134996	KP135259	KP134800	KP134919	MZ913688	Floudas and Hibbett, 2015; Chen et al., 2021
*Raduliporus aneirinus*	HHB-15629	USA	KP135023	KP135207	KP134795	—	—	Floudas and Hibbett, 2015
*Raduliporus aneirinus*	Wu 0409-199	China	MZ637068	MZ637267	—	OK136096	MZ913712	Chen et al., 2021
*Resiniporus pseudogilvescens*	Wu 9508-54	China	MZ637069	MZ637269	—	—	—	Chen et al., 2021
*Resiniporus pseudogilvescens*	Wu 1209-46	China	KY688203	MZ637268	MZ748436	OK136097	MZ913713	Chen et al., 2018; Chen et al., 2021
*Resiniporus resinascens*	BRNM 710169	Czech Republic	FJ496675	FJ496698	—	—	—	Tomšovský et al., 2010
*Trametopsis aborigena*	Robledo 1236	Argentina	KY655336	KY655338	—	—	—	Gómez-Montoya et al., 2017
*Trametopsis aborigena*	Robledo 1238	Argentina	KY655337	KY655339	—	—	—	Gómez-Montoya et al., 2017
*Trametopsis brasiliensis*	Meijer 3637	Brazil	JN710510	JN710510	—	—	—	Miettinena et al., 2012
*Trametopsis cervina*	Cui 18017	China	ON041041	ON041057	—	ON099414	ON083780	Liu et al., 2022c
*Trametopsis cervina*	Dai 21820	China	ON041044	ON041060	ON099407	ON099416	ON083783	Liu et al., 2022c
*Trametopsis cervina*	TJV-93-216T	USA	JN165020	JN164796	JN164839	JN164877	JN164882	Justo and Hibbett, 2011
*Trametopsis montana*	Cui 18363	China	ON041038	ON041054	ON099403	ON099411	ON083777	Liu et al., 2022c
*Trametopsis montana*	Cui 18383	China	ON041039	ON041055	ON099404	ON099412	ON083778	Liu et al., 2022c
*Trametopsis tasmanica*	Cui 16606	Australia	ON041048	ON041064	ON099409	ON099419	ON083787	Liu et al., 2022c
*Trametopsis tasmanica*	Cui 16607	Australia	ON041049	ON041065	ON099410	ON099420	ON083788	Liu et al., 2022c

Newly generated sequences for this study are shown in bold.

Additional sequences were downloaded from GenBank (**Table 1**). All sequences of ITS, nLSU, *RPB1*, *RPB2*, and *TEF1* were respectively aligned in MAFFT 7 (Katoh and Standley, 2013; http://mafft.cbrc.jp/alignment/server/) and manually adjusted in BioEdit (Hall, 1999). Alignments were spliced in Mesquite (Maddison and Maddison, 2017). The missing sequences and ambiguous nucleotides were both coded as “N.”

Most parsimonious phylogenies were inferred from the combined 2-gene dataset (ITS+nLSU) and 5-gene dataset (ITS+nLSU+*RPB1*+*RPB2*+*TEF1*), and their congruences were evaluated with the incongruence length difference (ILD) test (Farris et al., 1994) implemented in PAUP* 4.0b10 (Swofford, 2002) under heuristic search and 1,000 homogeneity replicates. Phylogenetic analyses followed Sun et al. (2020). In phylogenetic reconstruction, the sequences of *Phanerochaete albida* Sheng H. Wu and *P. alnea* (Fr.) P. Karst. obtained from GenBank were used as outgroups to root trees following Liu et al. (2022c). Maximum parsimony (MP) analysis was applied to the combined multiple gene datasets, and the tree construction procedure was performed in PAUP* version 4.0b10. All characters were equally weighted, and gaps were treated as missing data. Trees were inferred using the heuristic search option with TBR branch swapping and 1,000 random sequence additions. Max-trees were set to 5,000, branches of zero length were collapsed, and all parsimonious trees were saved. Clade robustness was assessed using a bootstrap (BT) analysis with 1,000 replicates (Felsenstein, 1985). Descriptive tree statistics tree length (TL), consistency index (CI), retention index (RI), rescaled consistency index (RC), and homoplasy index (HI) were calculated for each most parsimonious tree (MPT) generated. RAxmL v.7.2.8 was used to construct a maximum likelihood (ML) tree with a GTR+G+I model of site substitution including estimation of gamma-distributed rate heterogeneity and a proportion of invariant sites (Stamatakis, 2006). The branch support was evaluated with a bootstrapping method of 1,000 replicates (Hillis and Bull, 1993).

MrModeltest 2.3 (Posada and Crandall, 1998; Nylander, 2004) was used to determine the best-fit evolution model for the combined multigene dataset for Bayesian inference (BI). BI was calculated with MrBayes 3.1.2 with a general time-reversible (GTR) model of DNA substitution and a gamma distribution rate variation across sites (Ronquist and Huelsenbeck, 2003). Four Markov chains were run for two runs from random starting trees for 2.5 million generations (ITS+nLSU) and for 4 million generations (ITS+nLSU+*RPB1*+*RPB2*+*TEF1*), and trees were sampled every 100 generations. The first one-fourth generations were discarded as burn-in. A majority rule consensus tree of all remaining trees was calculated. Branches that received BT support for MP, ML, and Bayesian posterior probabilities (BPP) greater than or equal to 75% (MP and ML) and 0.95 (BPP) were considered as significantly supported. Trees were viewed in FigTree v1.4.4 (http://tree.bio.ed.ac.uk/software/figtree/). Sequence alignment was deposited at TreeBase (submission ID: 29921; http://www.treebase.org).

## Results

### Phylogeny

**Figure 1:**
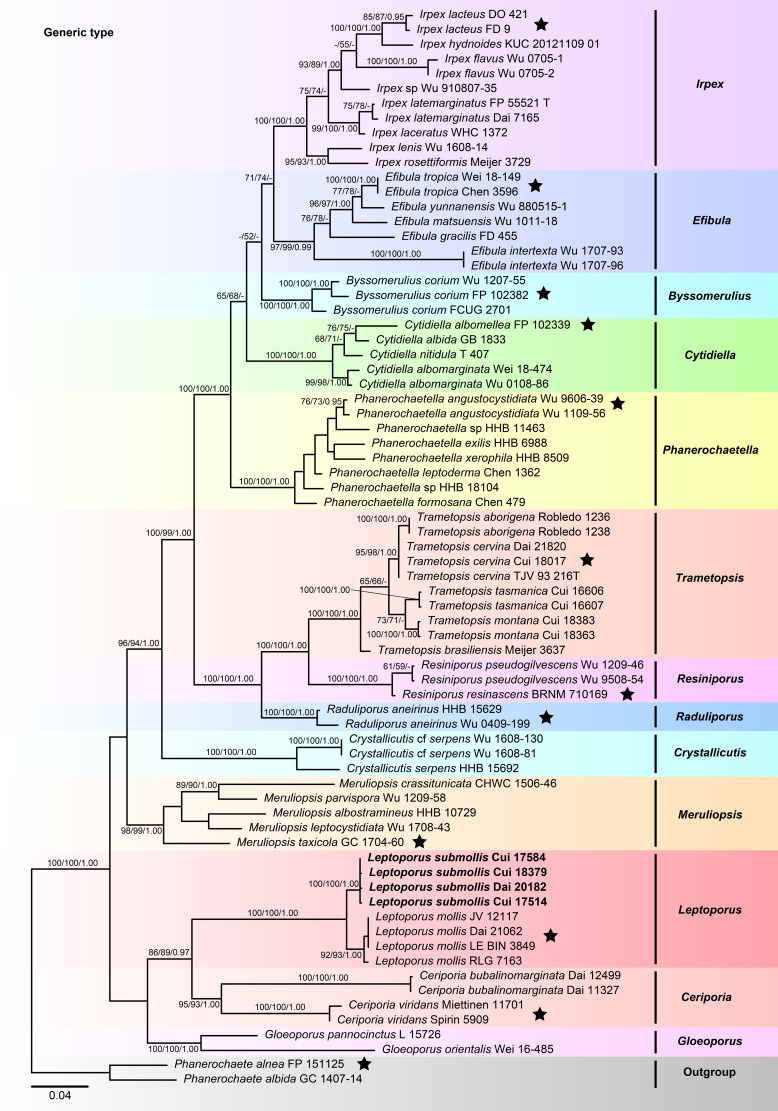
Maximum likelihood tree illustrating the phylogeny of Irpicaceae based on the combined sequence dataset of ITS+nLSU. Branches are labeled with maximum likelihood bootstrap higher than 50%, parsimony bootstrap proportions higher than 50%, and Bayesian posterior probabilities more than 0.90, respectively. Bold names = New species.

The combined 2-gene (ITS+nLSU) sequences dataset had an aligned length of 1,556 characters, including gaps (655 characters for ITS, 901 characters for nLSU), of which 998 characters were constant, 78 were variable and parsimony-uninformative, and 480 were parsimony-informative. MP analysis yielded 14 equally parsimonious trees (TL = 2,272, CI = 0.386, RI = 0.760, RC = 0.294, HI = 0.614). The best model for the concatenate sequence dataset estimated and applied in the BI was GTR+I+G with equal frequency of nucleotides. ML analysis resulted in a similar topology as MP and Bayesian analyses, and only the ML topology is shown in **Figure 1**.

**Figure 2:**
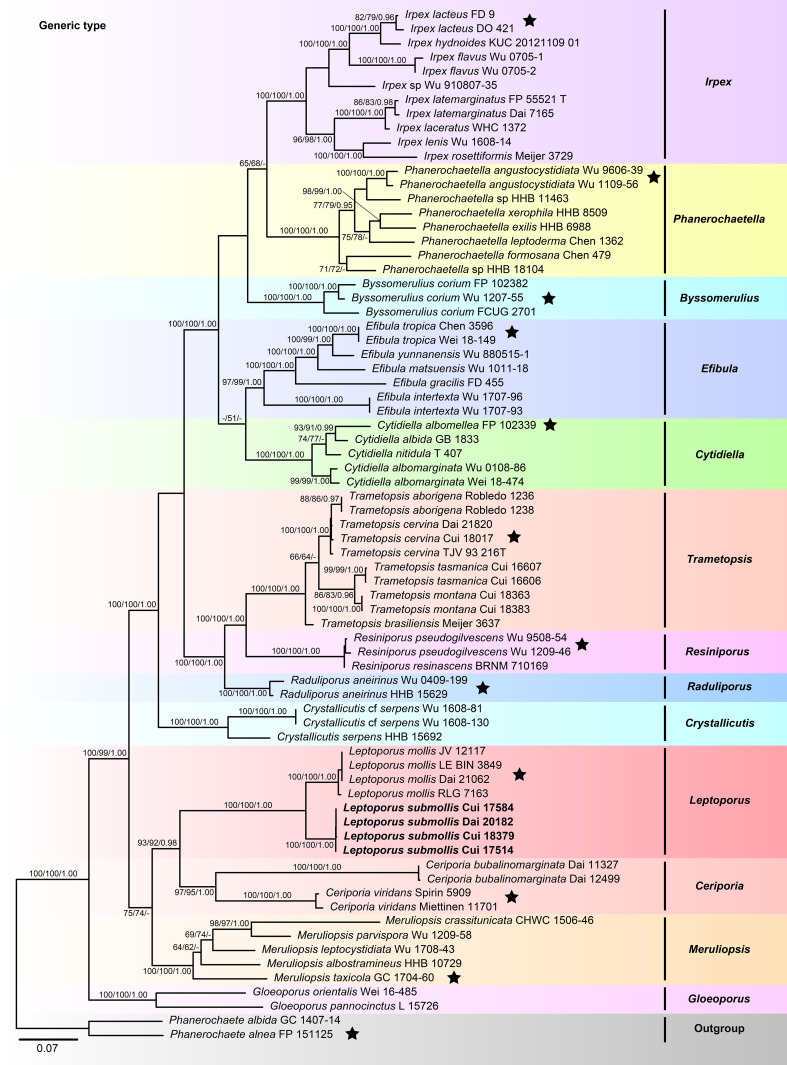
Maximum likelihood tree illustrating the phylogeny of Irpicaceae based on the combined sequence dataset of ITS+nLSU+*RPB1*+*RPB2*+*TEF1*. Branches are labeled with maximum likelihood bootstrap higher than 50%, parsimony bootstrap proportions higher than 50%, and Bayesian posterior probabilities more than 0.90, respectively. Bold names = New species.

The combined 5-gene (ITS+nLSU+*RPB1*+*RPB2*+*TEF1*) sequences dataset had an aligned length of 4,234 characters, including gaps (655 characters for ITS, 901 characters for nLSU, 1,192 characters for *RPB1*, 1,019 characters for *RPB2*, 467 characters for *TEF1*), of which 2,327 characters were constant, 207 were variable and parsimony-uninformative, and 1,700 were parsimony-informative. MP analysis yielded 33 equally parsimonious trees (TL = 10,223, CI = 0.332, RI = 0.665, RC = 0.221, HI = 0.668). The best model for the concatenate sequence dataset estimated and applied in the BI was GTR+I+G with equal frequency of nucleotides. ML analysis resulted in a similar topology as MP and Bayesian analyses, and only the ML topology is shown in **Figure 2**.

The combined datasets of ITS+nLSU and ITS+nLSU+*RPB1*+*RPB2*+*TEF1* contained sequences obtained from 74 fungal samples representing 45 taxa within the phlebioid clade (**Figures 1**, **2**). The phylogenetic trees (**Figures 1**, **2**) generated by MP, ML, and Bayesian analyses show that the new species *Leptoporus submollis* grouped with *L. mollis* with strong support (100% MP, 100% ML, 1.00 BPP; **Figures 1**, **2**) within Irpicaceae.

### Taxonomy

*Leptoporus* Quél., Enchiridion Fungorum in Europa media et praesertim in Gallia Vigentium: 175, 1886.

Type species: *L. mollis* (Pers.) Quél.

MycoBank: MB 17951

Basidiomata annual, effused-reflexed to pileate or resupinate, soft corky to corky or fragile. Pileal surface pale vinaceous to milky coffee, azonate, glabrous to tomentose. Pore surface flesh pink to snuff brown; pores circular to angular. Context pinkish buff to buff, corky. Tubes concolorous with pore surface, corky. Hyphal system monomitic; generative hyphae simple-septate, IKI–, CB–. Cystidia absent, cystidioles present. Basidiospores allantoid, cylindrical to oblong-ellipsoid, hyaline, thin-walled, smooth, IKI–, CB–. Causing a brown rot.

Specimen examined: *L. mollis*. BELARUS. Brestskaya Voblasts, Belavezhskaya Pushcha National Park, on stump of *Picea* sp., 19 October 2019, *Dai 21062* (BJFC 032721). CHINA. Heilongjiang, Yichun, Fenglin Nature Reserve, on fallen trunk of *Picea* sp., 5 August 2000, *Penttilä 13266* (IFP 014914). FINLAND. Koillissmaa, Oulanka National Park, on rotten wood of *Picea* sp., 17 September 1997, *Dai 2674* (IFP 014915).

**Figure 3:**
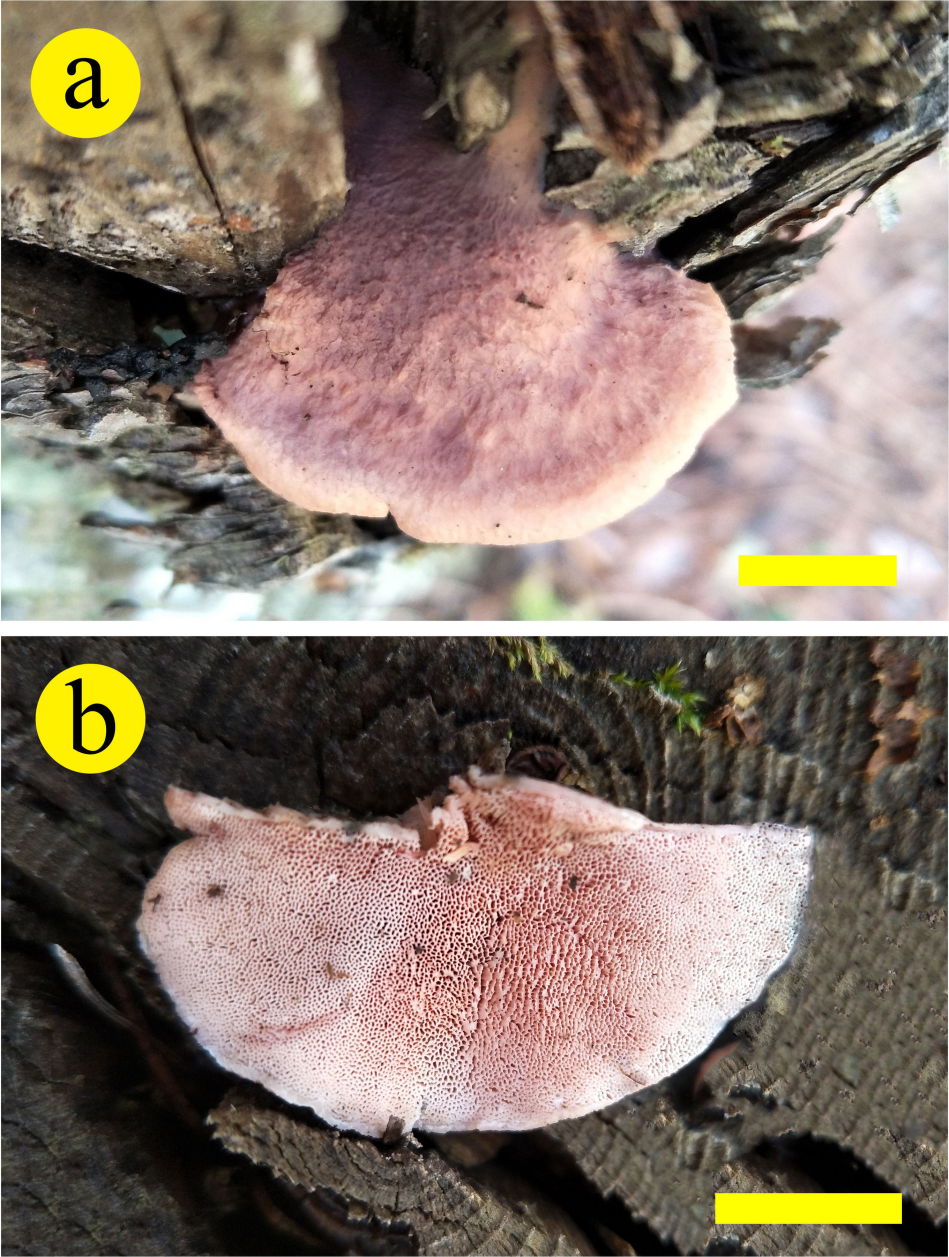
Basidiocarps of *Leptoporus submollis* (*Cui 17514*) (scale bar = 1.5 cm). Photo by Bao-Kai Cui.

**Figure 4:**
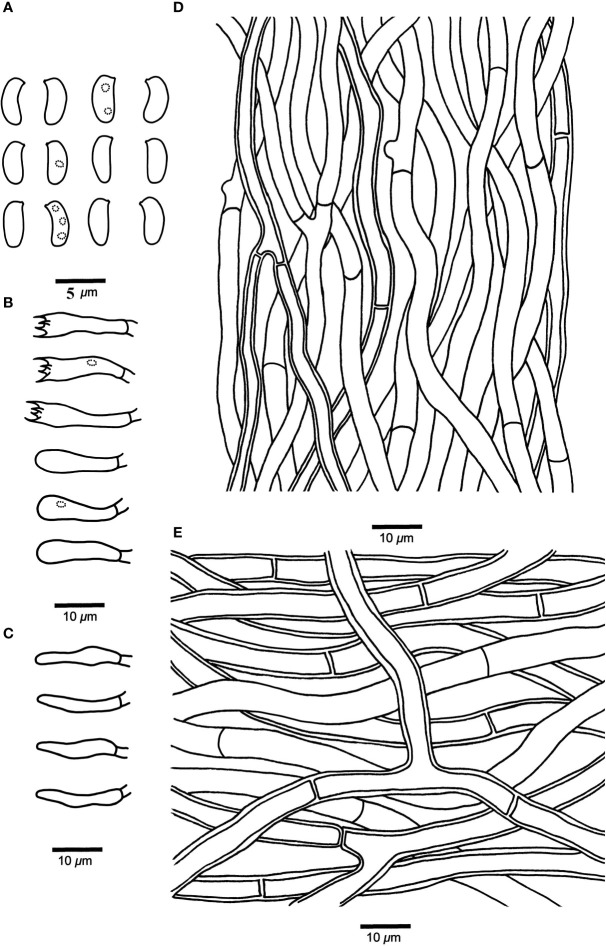
Microscopic structures of *Leptoporus submollis* (drawn from the holotype). **(A)** Basidiospores. **(B)** Basidia and basidioles. **(C)** Cystidioles. **(D)** Hyphae from trama. **(E)** Hyphae from context. Scale bar: **A** = 5 µm; **B–E** = 10 µm. Drawings by Shun Liu.

*Leptoporus submollis* B.K. Cui & Shun Liu, sp. nov. (**Figures 3**, **4**)

MycoBank: MB 840366

*Diagnosis. L. submollis* is characterized by its pale vinaceous to pale reddish pileal surface when fresh, becoming grayish brown to milky coffee upon drying, flesh pink to brownish vinaceous pore surface when fresh, becoming isabelline to snuff brown when dry, circular to angular pores (4–6 per mm) and cylindrical to oblong-ellipsoid basidiospores (4–4.8 μm × 1.8–2.3 μm).

*Type*. CHINA. Sichuan Province, Yanyuan County, on stump of *Pinus yunnanensis*, elevation 3,100 m, 15 August 2019, *Cui 17514* (holotype, BJFC 034373).

*Etymology*. “*submollis*” (Lat.) refers to the new species is similar to *L. mollis* in morphology.

*Fruiting body*. Basidiomata annual, effused-reflexed to pileate, solitary, soft corky, without odor or taste when fresh, corky and light in weight when dry. Pileus semicircular or irregular, projecting up to 2.5 cm, 5 cm wide, and 2 cm thick at base. Pileal surface pale vinaceous to pale reddish when fresh, becoming grayish brown to milky coffee upon drying, glabrous. Pore surface flesh pink to brownish vinaceous when fresh, becoming isabelline to snuff brown when dry; sterile margin narrow to almost lacking; pores circular to angular, 4–6 per mm; dissepiments slightly thick to thick, entire to lacerate. Context pinkish buff to buff, corky, up to 10 mm thick. Tubes concolorous with pore surface, corky, up to 6 mm long.

*Hyphal structure*. Hyphal system monomitic; generative hyphae simple-septate, IKI–, CB–; tissues unchanged in KOH.

*Context*. Generative hyphae hyaline, thin- to slightly thick-walled, occasionally branched, interwoven, 3.5–8.5 μm in diameter.

*Tubes*. Generative hyphae hyaline, thin- to slightly thick-walled, occasionally branched, 2–5 μm in diameter. Cystidia absent; fusoid cystidioles present, hyaline, thin-walled, 11–17 μm × 2–4 μm. Basidia clavate, bearing four sterigmata and a basal simple-septum, 12–20 μm × 3–5 μm; basidioles dominant, in shape similar to basidia, but smaller.

*Spores*. Basidiospores cylindrical to oblong-ellipsoid, hyaline, thin-walled, smooth, occasionally with 1–3 small oily inclusions, IKI–, CB–, 4–4.8 μm × 1.8–2.3 μm, L = 4.46 μm, W = 2.06 μm, Q = 2.02–2.13 (n = 90/3).

*Type of rot*. Brown rot.

*Additional specimens examined*. CHINA. Sichuan Province, Muli County, on stump of *Pinus yunnanensis*, elevation 3,050 m, 16 August 2019, *Cui 17584* (paratype, BJFC 034443). Xizang Autonomous Region (Tibet), Linzhi, on living gymnosperm tree, elevation 3,100 m, 18 July 2019, *Dai 20182* (paratype, BJFC 031853); Mangkang County, on stump of *Abies* sp., elevation 3,900 m, 8 September 2020, *Cui 18379* (paratype, BJFC 035238).

## Discussion

Decay mode is one of the most stable characteristics in Polyporales and has been used as the basis for distinguishing genera (Gilbertson and Ryvarden, 1986; Ryvarden, 1991). Among the Polyporales, nearly all of the brown-rot fungi species are clustered in the antrodia clade, which have been widely studied in recent years (Ortiz-Santana et al., 2013; Han et al., 2014; Shen et al., 2014; Song et al., 2014; Han et al., 2015; Han and Cui, 2015; Shen et al., 2015; Chen et al., 2015; Han et al., 2016; Chen and Cui, 2016; Song and Cui, 2017; Song et al., 2018; Shen et al., 2019; Liu et al., 2019; Liu et al., 2021a; Liu et al., 2021b; Liu et al., 2022a; Liu et al., 2022b; Liu et al., 2022d). In the phlebioid clade, most species can produce white-rot decay, with one notable exception, *L. mollis*, which can produce brown-rot decay (Binder et al., 2013; Chen et al., 2021). This result suggests that brown-rot fungi may have evolved more than once in Polyporales (Floudas and Hibbett, 2015).

In the present study, the phylogenetic analyses of Irpicaceae are inferred from the combined datasets of ITS+nLSU sequences (**Figure 1**) and ITS+nLSU+*RPB1*+*RPB2*+*TEF1* sequences (**Figure 2**). The results show that the genera of *Ceriporia* and *Leptoporus* grouped together and formed a highly supported lineage (**Figures 1**, **2**). Morphologically, *Ceriporia* spp. differs by possessing resupinate basidiomata, absence of cystidioles, and causing a white decay of wood (Chen et al., 2020; Chen et al., 2022). Therefore, *Ceriporia* and *Leptoporus* are treated as independent genera in Irpicaceae (Chen et al., 2020; Chen et al., 2021).

In our current phylogenetic analyses, *L. mollis* and *L. submollis* grouped together and formed a well-supported lineage (**Figures 1**, **2**). Morphologically, *L. mollis* may be confused with *L. submollis* by possessing annual growth habit, soft to corky basidiomata when fresh, and monomitic hyphal system with simple-septate generative hyphae, while *L. mollis* differs in having larger pores (2–4 per mm), narrower contextual generative hyphae (3–4 μm), and larger basidiospores (4.7–6 μm × 1.6–2.1 μm; Yu et al., 2004). Geographically, *L. mollis* has been reported in Asia, Europe, and North America (Gilbertson and Ryvarden, 1986; Ryvarden and Gilbertson, 1993; Núñez and Ryvarden, 2001; Yu et al., 2004). Yu et al. (2004) reported *Leptoporus* in China for the first time, which is distributed in Heilongjiang Province of China. In their study, the morphological characteristics of the studied specimens fit well with *L. mollis*. Therefore, there are two species of *Leptoporus* in China, viz., *L. mollis* is distributed in Northeast China, while *L. submollis* is distributed in Southwest China. In terms of ecological habits, *Leptoporus* species mainly grow on fallen trunk or stump of various coniferous trees (especially on *Abies* sp., *Picea* sp., and *Pinus* sp.) in the alpine plateau and cold temperate zone and cause a brown decay of wood.

### Nomenclature

BI, Bayesian inference; BJFC, Herbarium of the Institute of Microbiology, Beijing Forestry University; BGI, Beijing Genomics Institute; BPP, Bayesian posterior probabilities; BT, bootstrap; CB, Cotton Blue; CB–, acyanophilous; GTR+I+G, general time reversible+proportion invariant+gamma; IFP, Herbarium of the Institute of Applied Ecology, Chinese Academy of Sciences; IKI, Melzer’s reagent; IKI–, neither amyloid nor dextrinoid; ILD, incongruence length difference test; ITS, internal transcribed spacer; KOH, 5% potassium hydroxide; L, mean spore length (arithmetic average of all spores); ML, maximum likelihood; MP, maximum parsimony; MPT, most parsimonious tree; n (a/b), number of spores (a) measured from given number (b) of specimens; nLSU, large subunit of nuclear ribosomal RNA; Q, variation in the L/W ratios between the specimens studied; *RPB1*, DNA-directed RNA polymerase II subunit 1; *RPB2*, DNA-directed RNA polymerase II subunit 2; TL, tree length; W, mean spore width (arithmetic average of all spores); CI, consistency index; RI, retention index; RC, rescaled consistency index; TBR, tree-bisection-reconnection HI, homoplasy index; *TEF1*, translation elongation factor 1-α.

## Data availability statement

The datasets presented in this study can be found in online repositories. The names of the repository/repositories and accession number(s) can be found in the article/Supplementary Material.

## Author contributions

B-KC designed the research. B-KC, SL, Y-FS, XJ, C-GS and T-MX prepared the samples. SL, C-GS and T-MX conducted the molecular experiments and analyzed the data. SL, Y-FS and B-KC drafted the manuscript. All authors contributed to the article and approved the submitted version.
